# Consanguinity and Neonatal Death: A Nested Case-Control Study

**Published:** 2014-12

**Authors:** Reza Chaman, Mahshid Gholami Taramsari, Ahmad Khosravi, Mohammad Amiri, Kourosh Holakouie Naieni, Masoud Yunesian

**Affiliations:** 1 Department of Community Medicine, School of Medicine, YasujUniversity of Medical Sciences, Yasuj, Iran; 2 HealthDeputy, Yasuj University of Medical Sciences, Yasuj, Iran; 3 Center for Health Related social and Behavioral Sciences Research, School of Medicine, Shahroud University of Medical Sciences, Shahroud, Iran; 4 Department of Public Health, School of Public Health, Shahroud University of Medical Sciences, Shahroud, Iran; 5 Department of Epidemiology and Biostatistics, School of Public Health, Tehran University of Medical Sciences, Tehran, Iran; 6 Department of Environmental Health, School of Public Health, Tehran University of Medical Sciences, Tehran, Iran

**Keywords:** Neonatal Death, Nested Case- Control Study, Consanguinity, Rural Areas, Iran

## Abstract

**Objective:** Although numerous studies have found higher rates of abortion and still births following consanguinity (familial marriages), the question of whether consanguinity significantly increases the risk of neonatal death has inadequately been addressed.This study aims to evaluate familial marriage effects on neonatal death in rural areas in Iran.

**Materials and methods:** In this nested case-control study, 6900 newbornswho were born in rural areas of Kohgiluyeh and Boyerahmad Province (South-West of Iran)were followed till the end of neonatal period, and neonatal death was the outcome of interest. Subsequently 97 cases and 97 controls were selected in study cohort by using risk set sampling model. Crude and adjusted odds ratios (OR) were estimated by usinga conditional logistic regression model.

**Results:** In the final model, prematurity (OR = 5.57), low birthweight (LBW) (OR = 7.68), consanguinity (first cousins) (OR = 5.23), C-section (OR = 7.27), birth rank more than 3 (OR = 6.95) and birthsinterval less than 24 months (OR = 4.65) showed significant statistical association with neonatal mortality (p < 0.05).

**Conclusion:** According to our findings, after adjusting the effects of other significant risk factors, familial marriageto first cousins is considered asan important risk factor for neonatal death.

## Introduction

Neonatal mortality rate is one of the most important indicators forthe evaluation of children health status and improvement of this health indicator is quite gradual in rural areas of Iran (1). Consanguinity or inbreeding is defined as familal marriages and it is categorized as 'close consanguinity' or 'first cousins' and 'remote consanguinity' or 'second cousins' or ' distant relative marriages' (2-4). Several studies have shown deleterious effects of consanguinous marriages on abortion, still births, neonal deaths and infant mortalities (5-10). In several studies, consanguineous marriage has been reported as the most significant cause of genetically associated mortalites and related- by-blood couples were more likely to experience offspring death than non-familial couples (11,12). A research in Pakistan found that first cousin marriages were more prone to experience a child's death, compared to not-related-by-blood couples (13). Another study demonstrated a correlation between the genetic effect of consanguinity and offspring death among first cousin marriages after controlling for the non-genetic related factors (14).

In assessing the effects of consanguinity on neonatal and infant death, it is clearly accepted that variables such as maternal education, maternal age, birth intervals, gestational age and birth weight need to be adequately controlled (4).

Altough numerous studies have found higher rates of abortion and still births following consanguineous marriages, for countries such as Iran the question of whether consanguinity significantly increased the risk of neonatal death is inadequately addressed.This study was done to give an evidence-based answer to this question and to provide a better evaluation of familial marriage effects on neonatal death in rural areas in Iran. To achieve this aim, we tried to use a sophisticated design as well as modern analytic techniques.

## Materials and methods

This research was carried out as a nested case-control study and the study cohort included all of the neonates bornduring 12 months in rural areas of Kohgiluyeh and Boyrahmad province which is located in South-West of Iran. All of the cohort subjects were followed during the first 28 days of life and the outcome of interest was the neonatal death.For each case at the time of death, one control was randomly selected among all newborns with same birth date. According to the risk set sampling method, 97 controls were selected due to occurrenceof 97 cases of neonatal death in the 6900 studied cohort.The sample size was enoughto establish regression method analysis, based ongeneral rule of 5-10 subjects per variable in each comparison group.

The dependent variable was neonatal death andtheindependent variables were parents relation (first cousins vs. remote consanguinity and non-familial marriages), gender (malevs. female), gestational age (<37 weeksvs. ≥37 weeks), birth weight (<2500gr vs. ≥2500gr), maternal age (<18 or > 35 vs. ≥18 or ≤ 35), birth rank (>3 vs. ≤3), delivery route (C-section vs. normal vaginal delivery (NVD)) and birth spacing (<24 months vs. ≥ 24 months). The data were analyzed using univariate and multivariate conditional logistic regression methods in Stata software (Stata Corp, USA) version 10.

## Results

Based on descriptive results, there are moderate discrepancies in frequencies of consanguineous marriages between the two groups (Table 1). Comparing the two groups, great differences are seenbetweenLBW, prematurity, delivery type (C-section) and birth rank more than 3 of the two groups (Table 1). Univariate conditional logistic regression was performedto estimate crude ORs (Table 1).

In the next step, each risk factor with marked association (p value< 0.2) was selected for multivariate analysis. Thus, conditional logistic regression model included thebirth weight (OR = 9.8, 95% CI = 3.90-24.60), gestational age (OR = 8.8, 95% CI=3.50-22.20), parents relation (OR = 1.5, 95% CI=0.84-2.95), births spacing (OR = 1.79, 95% CI=.78-4.08), delivery route (OR = 2.8, 95% CI=1.36-5.76) and birth rank (OR = 1.8, 95% CI =0.96-3.38).

Final model includes following variables with significant statistical association (p value < 0.05): close consanguinity (adjusted odds ratio (AOR = 5.23), prematurity (AOR = 5.57), LBW (AOR = 7.68), C-section (AOR = 7.27), birth rank more than 3 (AOR = 6.95) and births spacing less than 24 months (AOR = 4.65) (Table 2).

## Discussion

This studyshows that first cousin marriages, prematurity, LBW,C-section, birth spacing less than 24 months and birth rank more than 3were potential risk factors for neonatal death.

In this study, an increased risk of neonatal death was found in the consanguineous group and this finding is consistent with previous similar studies in Iran (9,15). Also the observed association is in the same direction as that demonstrated by researchers on evaluation of consanguineous marriages and children mortality correlation (5-8,12,16).

In thisstudy, gender of neonate was not a risk factor which is consistent with resultsof a study done in Kurdistan Province of Iran. But delivery route (C-section) which did not have a significant effect in the above-mentioned study, revealed higher OR with significant statistical association in present study (17).

**Table 1:** Distribution of risk factors among cases and controls and ORs from univariate conditional logistic regression; a Nested Case-Control study in a rural part of Iran

Variables	Cases n (%)	Controls n (%)	OR (95%CI)
**Sex of neonate**			
** Female**	**43 (44.3%)**	**52 (53.6%)**	**1**
** Male**	**54 (55.7%)**	**45 (46.4%)**	**1.43 (0.82-2.50)**
**Birth weight **			
** ≥2500gr**	**46 (47.4%)**	**91 (91.8%)**	**1**
** <2500gr**	**51 (52.6%)**	**6 (8.2%)**	**9.8 (3.90-24.60)**
**Gestational age**			
** ≥37 weeks**	**50(51.5%)**	**89(91.8%)**	**1**
** <37 weeks**	**47(48.5%)**	**8(8.2%)**	**8.8 (3.50-22.20)**
**Delivery type**			
** NVD**	**66 (68%)**	**84 (86.6%)**	**1**
** C-section**	**31(32%)**	**13 (13.4%)**	**2.8 (1.36-5.76)**
**Birth rank:**			
** < 3**	**63 (64.9%)**	**75 (77.3%)**	**1**
** ≥ 3**	**34 (35.1%)**	**22 (22.7%)**	**1.8 (0.96-3.38)**
**Birth spacing**			
** ≥24 months**	**46 (47.4%)**	**56 (57.7%)**	**1**
** <24 months**	**19 (19.6%)**	**13 (13.4%)**	**1.79 (0.78-4.08)**
**First gestation**	**32 (33%)**	**28 (28.9%)**	**-**
**Maternal age**			
** ≥18 or ≤ 35**	**84 (86.6%)**	**88 (90.7%)**	**1**
** < 18 or > 35**	**13 (13.4%)**	**9 (9.3%)**	**1.5 (0.61-3.67)**
**Parents Relative**			
** Non-familial**	**54 (55.7)**	**60 (61.9)**	**1**
** Distant relatives**	**34 (35.1)**	**24 (24.7)**	**0.74 (0.30- 1.85)**
** First Cousins**	**9 (9.3)**	**13 (13.4)**	**1.5 (0.84- 2.95)**

**Table 2:** Results of Multivariate conditional logistic regression of neonatal mortality risk factors; a Nested Case- Control study in a rural part of Iran

Variables	Adjusted OR	95%CI	p value
**Birth weight**			
** ≥2500gr**	**1**	**-**	**-**
** <2500gr**	**7.68**	**1.49- 39.55**	**0.015**
**Gestational age**			
** ≥37 weeks**	**1**	**-**	**-**
** <37 weeks**	**5.57**	**1.12- 27.60**	**0.035**
**Consanguinity**			
** Unrelative**	**1**	**-**	**-**
** Second Cousin**	**0 .31**	**0.07- 1.4**	**0.126**
** First Cousin**	**5.23**	**1.59- 17.21**	**0.007**
**Birth rank **			
** ≤3**	**1**	**-**	**-**
** >3**	**6.95**	**1.90- 25.28**	**0.003**
**Delivery route **			
** NVD**	**1**	**-**	**-**
** C-section**	**7.27**	**2.05- 25.72**	**0.002**
**Birth Spacing **			
** ≥24 months **	**1**	**-**	**-**
** <24 months**	**4.65**	**1.13-19.13**	**0.033**

Our observations are similar to a case-control study which was conducted in Brazil and the results indicated that prematurity and LBWwere risk factors for neonatal death. They did not estimate significant OR for delivery route but we identified C-section as an important risk factor. Neither of the studies showed significant correlation between maternal age and neonatal death (18). In this research, theeffects of prematurity and LBW on neonatal mortalitywere similar tothe findings of a research performed by a group of investigators inthe city of Yazd in Iran,but contrary to our resultsregarding the effect of gender, they reported the determinant effect of gender. Present study shows that births spacing less than 24 months increases the risk of neonatal death but in that study birth intervals less than 12 months was identified as a potential risk factor (19).There were significant positive associations between neonatal death and prematurity, LBW, maternal age older than 35 years and birth rank higher than 5 in Shirvani and colleagues’ survey (20).

Numerousinvestigations have shown that LBW with or without prematurity plays in a complex causal framework of neonatal death, involving genetic and environmental factors related to socioeconomic status (21- 23). Thus, as a limitation of this research, it is focused on main risk factors of neonatal death and our suggestion is extension of nested case-control study to all probable maternal, neonatal and socioeconomic risk factors of neonatal mortality.

## Conclusion

The key findings of the present study is a significant positive association between close consanguinity and neonatal death, after controlling the effects of prematurity, LBW, C-section, birth spacing less than 24 months and birth rank more than 3.
